# Sulfoxaflor biodegradation: environmental fate, degrading microorganisms, and mechanisms of nitrile hydratase-mediated catalysis

**DOI:** 10.3389/fmicb.2026.1809475

**Published:** 2026-05-25

**Authors:** Shanshuo Mou, Fenghua Li, Jiaqiang Guo, Di Huang, Hongling Liu, Haibo Yuan, Yi Jiang, Tengfei Wang

**Affiliations:** 1 State Key Laboratory of Bio-based Material and Green Papermaking, Qilu University of Technology (Shandong Academy of Sciences), Jinan, Shandong, China; 2 Key Laboratory of Shandong Microbial Engineering, School of Bioengineering, Qilu University of Technology (Shandong Academy of Sciences), Jinan, Shandong, China; 3 Shandong Xinxibao Co., Ltd., Jinan, Shandong, China

**Keywords:** biodegradation, microorganisms, neonicotinoid insecticides, nitrile hydratase, sulfoxaflor

## Abstract

Sulfoxaflor (SXF), a novel sulfoximine insecticide, has been widely used as a substitute for traditional neonicotinoids (NEOs) to control piercing-sucking pests in agriculture. However, its environmental persistence, high mobility in water and soil, and potential toxicity to non-target organisms (e.g., honeybees, aquatic invertebrates) as well as the toxic risks of its long-lasting metabolites (e.g., X11719474) have raised significant ecological concerns. Biodegradation, as an environmentally friendly and efficient remediation strategy, plays a crucial role in regulating the environmental fate of SXF, and has become a research hotspot in recent years. This review systematically summarizes the environmental behaviors of SXF, including its migration, transformation, and residue characteristics in water bodies, soils, and agricultural products. It focuses on collating the reported SXF-degrading microorganisms (mainly bacteria such as *Ensifer*, *Pseudomonas*, *Aminobacter*, and cyanobacteria like *Synechocystis salina*) and their degradation efficiencies under different environmental conditions. Moreover, the review elaborates on the core role of nitrile hydratase (NHase) in SXF biodegradation, including the types, structural characteristics, and catalytic mechanisms of SXF-degrading NHases, as well as the key factors (environmental factors, structural residues, chemical modulators) influencing NHase catalytic activity. Additionally, biotechnological optimization strategies for enhancing SXF biodegradation efficiency, such as heterologous expression and immobilization of NHase, are discussed in detail. Finally, the current research gaps and future research directions are prospected, aiming to provide comprehensive theoretical support for the scientific application and environmental risk control of SXF, and offer references for the biodegradation research of novel NEOs and similar insecticides.

## 1 Introduction

Neonicotinoid insecticides (NEOs) emerged in the 1990s as fourth-generation pesticides following organophosphates, pyrethroids, and carbamates. As core agents for controlling sucking insect pests, these compounds have secured a significant share of the global insecticide market (Hladik et al., 2018; Sun et al., 2024). NEOs account for greater than 25% of global insecticide sales with thiamethoxam, imidacloprid and clothianidin comprising almost 85% of the total neonicotinoid sales in 2012 (Bass et al., 2015). Functioning as selective agonists, NEOs target insect nicotinic acetylcholine receptors (nAChRs) (Hussain et al., 2016). By selectively binding to insect-specific nAChR subunits and mimicking acetylcholine, NEOs induce persistent receptor activation and disrupt neurotransmission. This triggers an excessive influx of sodium ions, ultimately leading to insect paralysis and death (Jeschke et al., 2011). However, the widespread and excessive application of NEOs worldwide has triggered a series of environmental deteriorations in soil and water systems (Huang et al., 2024; Mohanty, 2024; Satheeshkumar et al., 2025). Furthermore, their long-term and large-scale use has accelerated the development of pest resistance (Matsuda et al., 2020). Additionally, due to their environmental persistence, high water mobility, and toxicity to non-target organisms (e.g., bees and birds), regulatory bodies such as the EU have imposed restrictions on certain traditional neonicotinoids (Goulson, 2013; Klingelhöfer et al., 2022).

**FIGURE 1:**
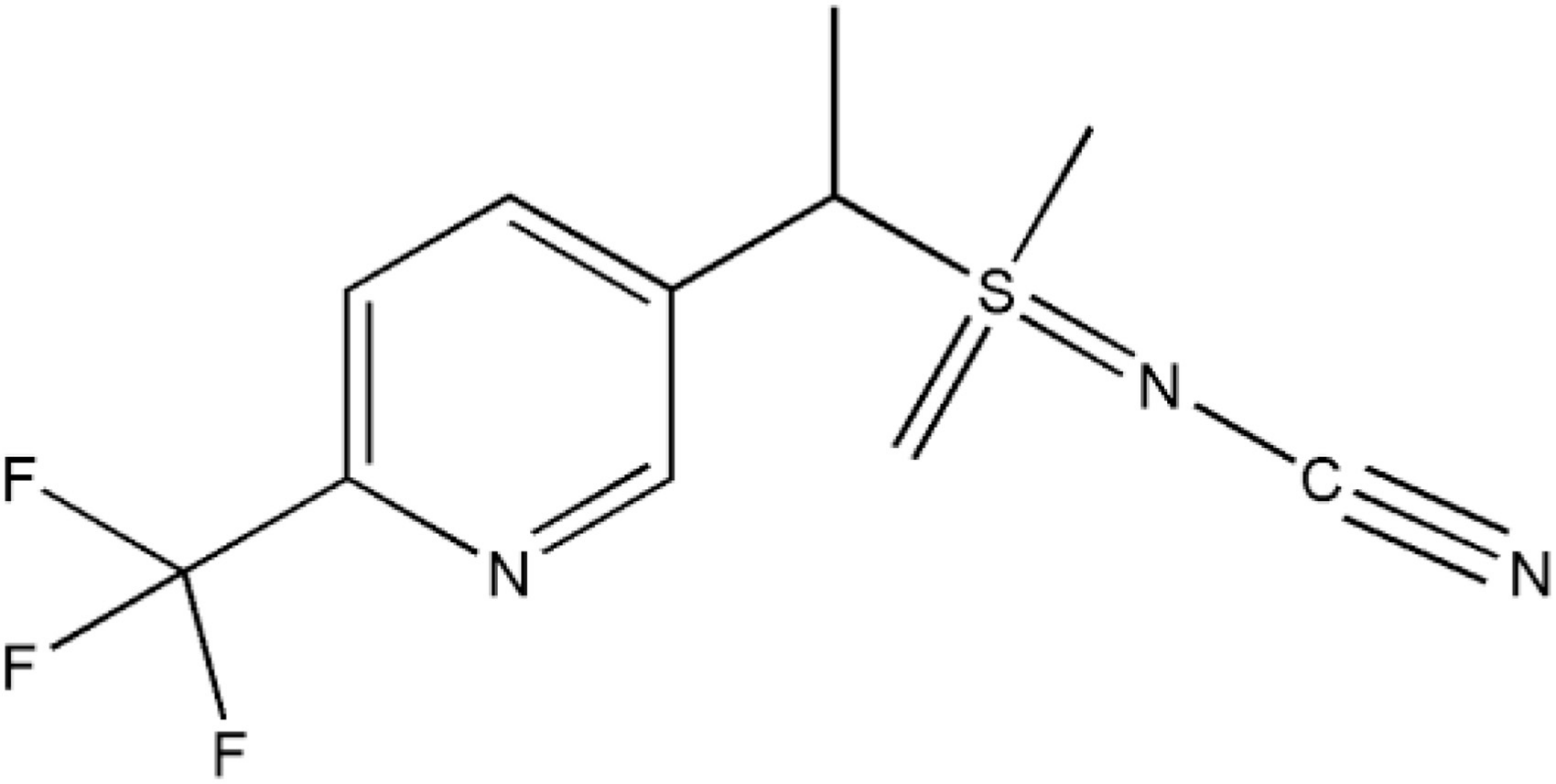
Structure formula of sulfoxaflor.

Sulfoxaflor (N-[methyloxido[1-[6-(trifluoromethyl)-3-pyridinyl] ethyl]-λ4-sulfanylidene] cyanamide), SXF was commercialized as a replacement for neonicotinoids (NEOs), which exhibit high acute toxicity to pollinators, and is classified as a member of sulfoximines—a novel class of insecticidal agents (Sparks et al., 2013). Developed by Dow Agro Sciences, the chemical structure of SXF is illustrated in Figure 1 below. SXF is recognized by the Insecticide Resistance Action Committee (IRAC) as a Group 4C insecticide, distinct from NEOs classed in Group 4A (Kramer and Solomon, 2025). First registered with the US Environmental Protection Agency (EPA) in 2010 (Centner et al., 2018), SXF subsequently received approval for use in Canada, Australia, and other regions, and has now been registered in 81 countries (Siviter et al., 2020). SXF has been widely used in controlling the pests including *Aphis gossypii* Glover, *Philaenus spumarius*, and *Laodelphax striatellus* on crops such as brown rice and lettuce (Chen et al., 2016; Chung et al., 2017; Dáder et al., 2019; Kim et al., 2017; Yang et al., 2025; Zhang et al., 2016). The unique chemical structure of SXF (containing a sulfoximine functional group) enables it to evade cross-resistance in pest strains, maintaining high efficacy against pest populations resistant to traditional NEOs, while exhibiting lower mammalian toxicity and better environmental compatibility (Chen et al., 2025; Convertini, 2018; Watson et al., 2021; Zhu et al., 2011). However, SXF will exert adverse effects on honeybees (Siviter et al., 2018), causing significant mortality by altering GST (glutathione S-transferase) activity, and inducing histological damage in the brain, hypopharyngeal gland, and midgut (Ibrahim et al., 2023). Moreover, SXF poses risks of migration and diffusion in the environment due to its high water solubility and low soil affinity (Jiang H. et al., 2023; Naumann et al., 2022). The residues and degraded products of SXF in soil and water may affect animals and microorganisms in the soil and water bodies (Brooks et al., 2021; Gauthier and Mabury, 2021; Zhang et al., 2020), and the ecotoxicity of its degradation products has also attracted increasing attention (Gauthier and Mabury, 2021; Yuan et al., 2023). Therefore, clarifying its environmental fate and efficient degradation pathways is of great significance.

Extensive strategies have been developed for the elimination of SXF residues. The degradation pathways of SXF are primarily divided into two categories, physical-chemical and biological degradation. Various physicochemical methods have been reported including photodegradation under UV light (Yang et al., 2022; Zhao et al., 2023), advanced oxidation processes (Bhat et al., 2022; Raashid et al., 2023) and physical adsorption (Alharthi et al., 2025). Although technology can achieve short-term degradation of pollutants, there are limitations such as strict reaction conditions, high costs, and the possibility of causing secondary pollution with photo-enhanced toxicity (Fan et al., 2023; Luo et al., 2025; Manonmani et al., 2023; Zhao Y. et al., 2025). Biodegradation, the primary natural purification approach for environmental pollutants, offers the advantages of high efficiency, mild reaction conditions and environmental benignity, and has thus emerged as a research hotspot for NEOs pollution remediation (Gautam and Dubey, 2023). Within bioremediation technologies, SXF can be degraded by microorganisms such as bacteria and fungi. So far, various SXF-degrading microorganisms have been isolated from the soil, water bodies, etc., covering bacterial and fungal species. Environmental degradation of SXF occurs mainly via oxidative, reductive and hydrolytic pathways (Zhao Y. et al., 2025), where nitrile hydratase (NHase, EC 4.2.1.84) exerts a crucial role in the bioconversion of SXF into its amide metabolites (Sun et al., 2021a, b). NHase plays a core role in the degradation of insecticides containing acrylate functional groups, and has been confirmed in the biodegradation studies of nicotine-based insecticides such as acetamiprid, thiacloprid, imidacloprid and flonicamid (Guo et al., 2021; Satheeshkumar et al., 2025; Sun et al., 2016, 2017, 2021b), providing important references for the biotransformation of SXF.

This review focuses on SXF biodegradation. Based on systematically elaborating its environmental behaviors and toxicological properties, it emphasizes collating the reported resources of degrading microorganisms and the action mechanisms of key functional enzymes, analyzes the factors affecting biodegradation efficiency, and prospects application directions including enzyme engineering modification and microbial preparation development. The review aims to provide comprehensive theoretical support for the scientific application and environmental risk control of SXF, and meanwhile offer references for the research on biodegradation of novel NEOs. Compared with the two previously published related reviews (one focusing on the industrial application of NHase and its role in acetamiprid and thiacloprid degradation, and the other focusing on the environmental fate, degradation mechanism and toxic effects of SXF), this review achieves innovative breakthroughs in the following three aspects. Firstly, it constructs a systematic framework integrating “environmental behavior–degrading microorganisms–key enzymes–biotechnological optimization,” filling the gap of insufficient integration of multi-dimensional research on SXF biodegradation in existing studies. Secondly, it focuses on the core role of NHase, deeply dissects its classification, structural characteristics, catalytic mechanism and regulatory factors in SXF degradation, and supplements the lack of in-depth analysis of key enzyme systems in previous reviews. Thirdly, it highlights the practical application orientation, details biotechnological optimization strategies such as heterologous expression and immobilization of NHase, and provides actionable technical references for the industrialization of SXF bioremediation, which is rarely involved in previous reviews.

## 2 Environmental fate of SXF

**FIGURE 2:**
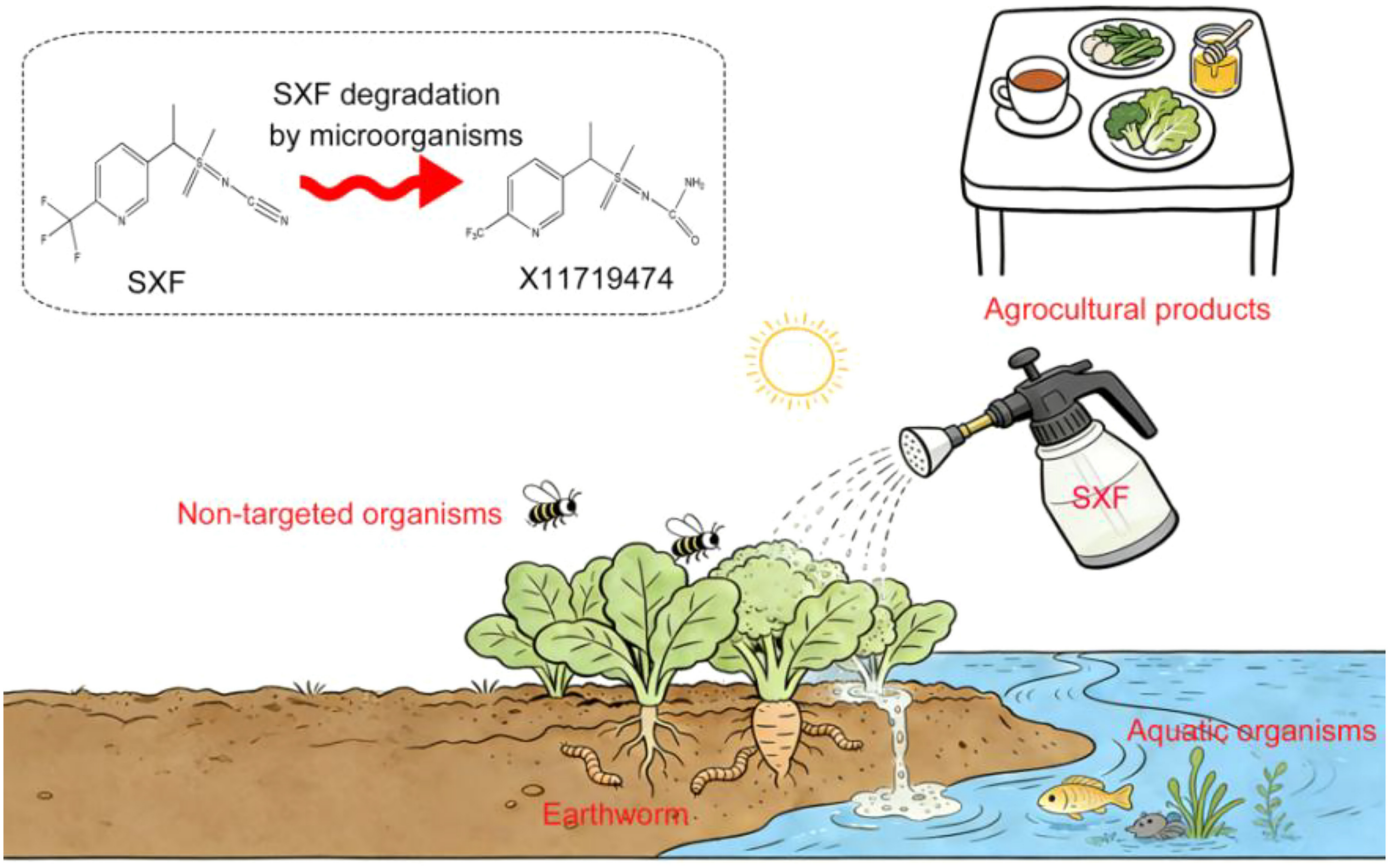
Illustration of the environmental behavior and toxicity of sulfoxaflor.

The environmental behavior of SXF is governed by its physicochemical properties and agricultural application patterns. SXF is commonly applied by foliar spraying and seed coating, with 22% of the active ingredient entering soil via foliar application and 61% via seed coating. Owing to its low soil sorption coefficient, up to 94% of SXF in soil can be transported into aquatic environments through groundwater leaching and surface runoff (Gauthier and Mabury, 2021). Its environmental distribution, degradation kinetics, and metabolite formation differ substantially among water, soil, and agricultural commodities. As illustrated in Figure 2, these migration pathways (rainfall leaching, surface runoff, and atmospheric deposition) and their subsequent impacts on soil and aquatic ecosystems are integral to understanding SXF’s overall environmental behavior and associated ecological risks.

### 2.1 SXF in water bodies

SXF enters aquatic environments primarily via spray drift, surface runoff, and subsurface leaching, leading to widespread detection in surface water, groundwater, and drinking water sources. In Chinese aquatic systems, detected concentrations reach 130.25, 157.66, and 58.9 ng/L in southern waters, urban rivers, and the lower Yangtze River, respectively (Tsegay et al., 2024). Globally, peak concentrations in surface water and groundwater range from 0.04 to 269 μg/L and 58–211 μg/L, posing risks to aquatic organisms (Morrissey et al., 2015; Thompson et al., 2020), SXF is highly photostable, with a photolytic half-life exceeding 1,000 days. Under aerobic conditions, the half-life in water is 11–65 days, whereas it extends to 84–261 days under anaerobic conditions (Thompson et al., 2020). The primary metabolite X11719474 is exceptionally persistent, with a half-life of up to 14 years in aquatic systems, creating a high risk of long-term accumulation (Cornelisse et al., 2019; Thompson et al., 2020). The high water solubility and long persistence of SXF directly increase its bioavailability for microbial uptake and NHase-catalyzed hydration (Section “4.2 Classification and structural characteristics of SXF-degrading NHases”). The failure to further degrade X11719474 leads to long-term ecological risk.

### 2.2 SXF in soils

SXF exhibits low sorption and high mobility in soil, with adsorption coefficients of 0.19–1.29 and organic carbon adsorption coefficients of 12–72, indicating weak retention by soil particles (US Environmental Protection, 2013). SXF degrades significantly faster under aerobic conditions than under anaerobic conditions, regardless of soil type or geographic region. Degradation rates vary across climatic and edaphic conditions. Field experiments in Shandong, Henan, and Zhejiang provinces in China showed that the half-life of SXF in cucumber fields was 1.5–7.2 days (Xu et al., 2012). In soils from the United States and Europe, SXF degraded rapidly under aerobic conditions with a half-life of only 0.05–0.6 days, while under anaerobic conditions, the half-life ranged from 0.17 to 2.5 days (Zhao Y. et al., 2025). The main metabolite X11719474 also degrades much more slowly than the parent compound, with a half-life of 320–532 days under aerobic conditions (Australian Pesticides and Veterinary Medicines Authority, 2013). In Europe, further degradation of SXF in soil generates secondary metabolites X11579457 and X11519540, with half-lives of up to 96–670 days and over 71 days, respectively (some exceeding 1,000 days).^1^ The accumulation of these long-residual metabolites may exacerbate soil ecological risks. Regional differences in SXF half-lives may be related to soil texture and microbial community structure. Although anaerobic environments slow SXF degradation, they promote the formation of persistent metabolites, providing a basis for regionalized risk management. Soil pH, redox status, and microbial community structure directly regulate NHase expression and activity (Section “4.3 Key influences on catalytic activity of NHase”), which explains the large half-life differences among regions.

### 2.3 SXF in agricultural products

After foliar spraying, SXF and its metabolites can be widely residual in crops. Previous studies have reported the residues of SXF in agricultural products such as Puer tea (Liu et al., 2020), broccoli (Chen et al., 2022), *Cirsium japonicum*, *Cirsium maackii*, *Olea europaea* L. (Choi et al., 2025), and floral nectar (Zhou et al., 2022). The parent compound is the main residual form in the edible parts of crops, accounting for 16%–71% of total radioactive residues, while the metabolite X11719474 accounts for 7%–30% (European Food Safety Authority, 2015). Residue profiles differ among plant species; SXF, X11719474, and X11721061 are detected in rice, straw, and paddy water, whereas X11719474 comprises 31% of total radioactive residues in lettuce, exceeding the parent compound (17%) (European Food Safety Authority, 2015; Kim et al., 2017). Other metabolites including X11596066 and X11579457 have also been detected in planting systems. SXF residues remain stable for at least 24 months in oranges, peaches, wheat, soybeans, and other agricultural commodities stored at −20 °C (Australian Pesticides and Veterinary Medicines Authority, 2013). More importantly, SXF and its metabolites can be transferred to livestock via feed. X11719474 is poorly metabolized in lactating goats and laying hens, and mainly accumulates in feces, milk, eggs, and tissues, where it can persist stably for 45 days (Cho et al., 2022). Since X11719474 exhibits higher toxicity than the parent compound, it poses potential risks to food safety. Current data clearly illustrate the residue characteristics of SXF and its metabolites in crops and livestock-derived products, but maximum residue limits (MRLs) for different crops and long-term dietary risk assessments still need to be improved. In particular, the toxic amplification effect of persistent metabolites requires that both parent SXF and its metabolites be considered in food safety standard formulation, and full-chain residue monitoring should be strengthened.

## 3 Microorganisms for SXF degradation

Microbial degradation plays a pivotal role in the environmental fate of SXF, exerting a profound influence on the persistence of the parent compound and the formation, transformation, and environmental behavior of its metabolites. As an environmentally friendly and cost-effective strategy for eliminating pesticide residues, microbial degradation has garnered extensive attention in the remediation of SXF-contaminated environments. The core mechanism underlying microbial SXF degradation primarily involves the catalytic action of NHase, which facilitates the conversion of nitrile-containing organic compounds into their corresponding amides (Zhao Y. et al., 2025). A variety of prokaryotic microorganisms, including bacteria and cyanobacteria, have been identified to possess this metabolic capacity (Łukaszewicz et al., 2023). Table 1 summarizes the reported microorganisms capable of degrading SXF and their specific degradation effects.

**TABLE 1:** Reported microorganisms capable of degrading sulfoxaflor and their degradation effects.

Strains	Classification	Sources	Conditions	Effects	References
*Pseudomonas stutzeri* CGMCC 22915	*Pseudomonas stutzeri*	Soil samples collected from Nanjing, Jiangsu Province, China	Resting cells: 30 °C, 220 rpm, pH 7.0 (PBS); soil: 30 °C, 60% humidity; surface water (free cells): 30 °C, 220 rpm; surface water (immobilized cells): 30 °C, 220 rpm (calcium alginate beads)	Resting cells: 58.2% degradation of 814.28 μmol/L SXF in 2 h; soil: 65.6% degradation of 322.56 μmol/kg SXF in 3 days; surface water (free cells): 55.4% degradation of 84.53 μmol/L SXF in 4 days; surface water (immobilized cells): 72.7% degradation in 24 h; recombinant *E. coli* expressing PsNHase: 90.4% degradation of 798.29 μmol/L SUL in 5 min	Jiang et al., 2022
*Ensifer meliloti* CGMCC 7333	*Ensifer* genus, α-proteobacteria	Laboratory-preserved strain	Resting cells: OD600 = 5, 30 °C, 220 rpm, 96 h, 200 mg/L sulfoxaflor in 50 mmol/L PBS (pH 7.0) with 0.1 mmol/L CoCl_2_; Soil: 30 °C, 35% humidity, dark, 80 mg/kg sulfoxaflor, inoculum OD600 = 10 (2 mL); Recombinant *E. coli*/RHase: 30 °C, 800 rpm, 200 mg/L sulfoxaflor in 50 mmol/L PBS (pH 7.0)	Soil: 83.9% transformation in 9 days (half-life 3.4 days); purified NHase: 96.1% transformation in 120 min	Yang et al., 2021
*Aminobacter* sp. CGMCC 1.17253	*Aminobacter* genus	Soil samples collected from Kunming, Yunnan Province, China	Optimal pH 7.0 (stable in neutral/alkaline environments); optimal temperature: 40 °C (sensitive to high temperatures>40 °C); enhanced by Mn^2+^, Mg^2+^, Co^2+^,Ca^2+^, and EDTA; Inhibited by Cu^2+^, Zn^2+^, Ni^2+^ and organic solvents	Resting cells: 39.6% degradation of SXF (703.3 to 425.0 μmol/L) in 96 h; 94.4% molar conversion to X11719474; half-life = 5.5 days; Soil environment: 59.1% degradation in 9 days (half-life = 6.97 days), vs. 20.2% in non-inoculated soil; recombinant *E. coli* (expressing NHase): 68.35% conversion to X11719474 in 10 min	Yang et al., 2021
*Ensifer adhaerens* CGMCC 6315	Nitrogen-fixing, plant growth-promoting bacterium	Isolated by the research team; preserved in CGMCC	30 °C, 220 rpm; free cells in PBS (pH 7.0); immobilized in calcium alginate beads	Free cells: 77.91% removal of 0.89 mmol/L SXF in 3 h; immobilized cells: 78.57% removal in 2 h, near-complete elimination in 3 h (surface water)	Zhao Y.-X. et al., 2025
*Pseudaminobacter salicylatoxidans* CGMCC 1.17248	*Pseudaminobacter* genus	Soil	Resting cells: 30 °C, 220 rpm, pH 7.5 (PBS buffer); free cells in surface water: 30 °C, 220 rpm, pH 7.72; immobilized cells (calcium alginate): 30 °C, 150 rpm, pH 7.72; enzymes (AnhA/AnhB): optimal pH 7.0; AnhA optimal temp 50 °C, AnhB optimal temp 40 °C	Resting cells: 96.4% degradation of 0.83 mmol/L SXF in 30 min; free cells in surface water: 94.7% degradation in 1 h; immobilized cells: 82.8% degradation in 90 min, nearly complete degradation in 3 h; AnhA: Vmax = 4.40 U/mg, Km = 0.95 mmol/L; AnhB: Vmax = 0.41 U/mg, Km = 1.12 mmol/L	Zhao et al., 2023
*Ensifer* sp. DA6	*Ensifer* genus	Saline-alkaline soil from Binzhou, Shandong Province, China	Temperature: 30 °C (for resting cells); 45 °C (optimal for NHase); pH Neutral (pH 7.0, optimal for NHase); Co-factor: Co^2+^ required; immobilization: sodium alginate-CaCl_2_ gel beads	Resting cells (with Co^2+^): 62.13% degradation in 4 days (single substrate); 57.48% in 4 days (co-existing with Acetamiprid); half-life 2.84 days (single)/4.00 days (co-existing); immobilized cells: 28.33% degradation in Yellow River surface water (8 days, co-existing with acetamiprid); half-life 16.65 days; recombinant *E. coli* (overexpressing DA6 NHase): 68.35% degradation in 10 min (single); 40.07% in 10 min (co-existing with acetamiprid)	Yang et al., 2025
*Synechocystis salina*	Unicellular bloom-forming cyanobacteria	Culture Collection of Northern Poland (CCNP), Institute of Oceanography, University of Gdańsk, Poland	14-day incubation; SFX concentrations: 10 μg⋅L^1^ and 10 mg⋅L^1^; 22 ± 0.5 °C; 16/8 h day/night regime; continuous aeration; light intensity 70–130 μmol⋅m^2^⋅s^1^; BG 11 medium (pH 7.1–7.5)	7.6% SFX decline at 10 μg/L (M474: 436 ng/L^1^); 21.3% SFX decline at 10 mg/L (M474: 514 μg/L); 15.5% unknown metabolites	Łukaszewicz et al., 2023
*Microcystis aeruginosa*	Unicellular bloom-forming cyanobacteria	Culture Collection of Northern Poland (CCNP), Institute of Oceanography, University of Gdańsk, Poland	14-day incubation; SFX concentrations: 10 μg⋅L^1^ and 10 mg⋅L^1^; 22 ± 0.5 °C; 16/8 h day/night regime; continuous aeration; light intensity 70–130 μmol⋅m^2^⋅s^1^; Z8 medium (pH 7.1–7.5)	14.3% SFX decline at 10 μg/L (M474: 282 ng/L); 3.0% SFX decline at 10 mg/L (M474: 317 μg/L); full molar balance achieved	Łukaszewicz et al., 2023
*E. coli* pET28a-PsNHase-PsCbiM	*E. coli* (engineered strain)	Recombinant strain constructed by cloning PsNHase and PsCbiM genes from *Pseudomonas stutzeri* CGMCC 22915 into *E. coli* Rosetta (DE3)	Immobilized with calcium alginate; optimal initial concentration: 455.01 μmol/L; optimal pH 8.0; optimal temperature: 40 °C; reaction medium: Tris-HCl buffer (pH 7.0, 50 mmol/L)	Degrades sSXF to its amide via hydration; Half-life: 33–46.2 min (varies with initial concentration); retains 42.2% activity after 6 days of storage at 4 °C; reusable for 8 batches with 77.6% residual activity	Jiang H. et al., 2023

Microbial degradation is a pivotal process regulating the environmental fate of SXF, driven by diverse prokaryotes including bacteria and cyanobacteria. These microorganisms mediate SXF transformation primarily through hydration of its cyanamide functional group, catalyzed by NHase, producing the main metabolite N-(methyl(oxido){1-[6-(trifluoromethyl)pyridin-3-yl]ethyl}-λ4-sulfanylidene)urea (X11719474, which is also numbered as M474) (Kim et al., 2017). All reported SXF-degrading strains function via the same core mechanism of NHase-mediated hydration of the cyano group to form X11719474. This unified pathway explains why all strains produce the same dominant metabolite. X11719474, the amide metabolite of SXF, persists in crops (notably rotational and leafy ones) with residues occasionally exceeding the limit of quantification (LOQ), requiring risk mitigation for specific uses (EFSA et al., 2022). A critical note is that the metabolite X11719474 exhibits higher aquatic toxicity to aquatic invertebrates (e.g., *Daphnia magna*) than the parent compound SXF (Łukaszewicz et al., 2023). Degradation efficiency of SXF varies across microbial taxa, with bacterial strains generally exhibiting higher activity than cyanobacteria, and performance influenced by environmental matrices such as water, soil, and nutrient availability.

Bacteria are the most well-characterized SXF-degrading microorganisms, with key strains belonging to *Ensifer*, *Pseudomonas*, and *Aminobacter* genera. *Ensifer meliloti* CGMCC 7333 degrades 89.36% of 200 mg/L SXF in 96 h (half-life 1.2 days) and 83.9% of 80 mg/kg SXF in soil within 9 days (Yang et al., 2021). *Pseudomonas stutzeri* CGMCC 22915 shows exceptional efficiency, degrading 58.2% of 814.28 μmol/L SXF in 2 h (half-life 1.6 h) via a unique NHase (Jiang et al., 2022). *Aminobacter* sp. CGMCC 1.17253 mediates SXF hydration with a 39.6% degradation rate over 96 h and accelerates soil degradation, reducing SXF half-life from 27.68 to 6.97 days (Yang et al., 2020). *Ensifer* sp. DA6 uniquely co-degrades SXF and acetamiprid, retaining 57.48% SXF degradation efficiency in co-contamination scenarios (Yang et al., 2025).

Cyanobacteria such as *Synechocystis salina* and *Microcystis aeruginosa* contribute to aquatic SXF degradation but at lower rates, transforming 7.6–21.3% and 3.0%–14.3% of SXF (10 μg/L to 10 mg/L) over 14 days, respectively (Łukaszewicz et al., 2023). Key factors influencing microbial degradation include pH (optimal 6.0–7.0), temperature (30–45 °C), and cobalt availability (essential for NHase activity). Cell immobilization (e.g., calcium alginate entrapment) enhances microbial stability and reusability, while genetic engineering (e.g., recombinant *E. coli* expressing NHase and cobalt transporter genes) further improves degradation efficiency. The concentration of sulfate iron (SXF) in the environment is usually at the microgram level in water, but the concentration ranges used in most laboratory studies are higher than the actual field levels. When translating laboratory results to the field, this concentration difference must be taken into account. The activities, influencing factors, heterologous expression and immobilization of nitrile hydratase will be reviewed in detail in the next chapter.

**FIGURE 3:**
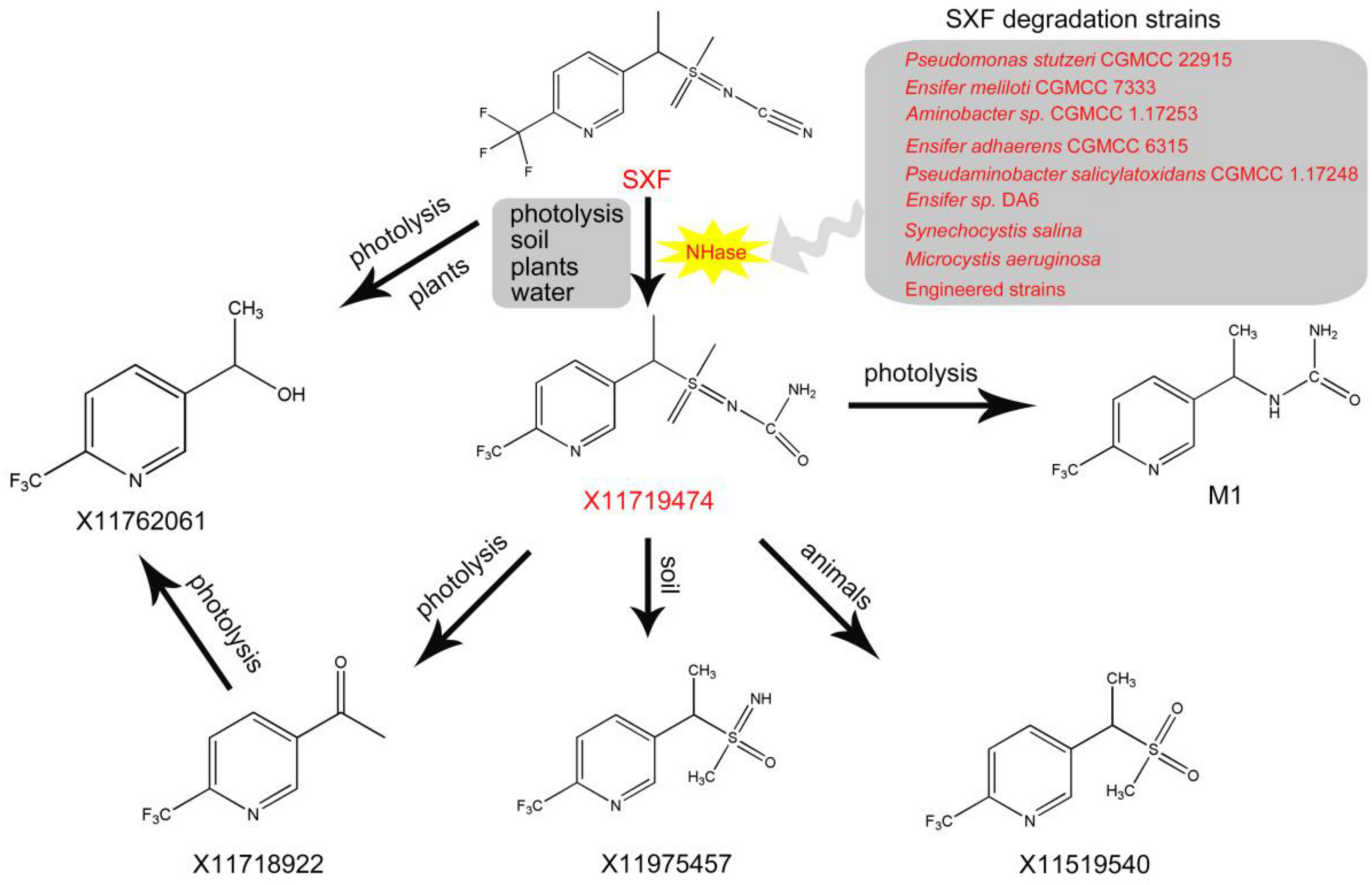
Sulfoxaflor degradation pathway.

In addition to the degradation of SXF mediated by microbial NHase, other degradation pathways of SXF have also been reported, as shown in Figure 3. SXF can be photodegraded to X11762061. The degradation amide product X11719474 of SXF can also be photodegraded to M1 and X11718922, where X11718922 is further photodegraded to X11762061; X11719474 can also be photodegraded to X11975457 and 11519540 in soil and animals, respectively. To date, the only confirmed biodegradation reaction of SXF is the NHase-mediated hydration of the cyano moiety to form the amide metabolite X11719474. No further biodegradation or mineralization of X11719474 has been reported in the literature. This restricted pathway reflects the current research status and is clearly presented in the degradation scheme (Figure 3), which distinguishes biotic transformations from abiotic processes such as photolysis.

Most of the high degradation efficiencies reported in the literature are derived from optimized laboratory conditions, including elevated substrate concentrations, pure strain culture, sufficient nutrient supply, and suitable pH and temperature settings. When extrapolated to actual field environments, such results are often constrained by multiple factors. First, the concentration of SXF used in laboratory studies is usually much higher than the residual level in the actual environment, which tends to overestimate the degradation rate. Second, complex matrices such as soil organic matter, coexisting pollutants and heavy metal ions will inhibit the activity of NHase to varying degrees. In addition, the inoculated functional strains are difficult to stably colonize in the open environment due to the competition of indigenous microorganisms. Moreover, the main metabolite X11719474 is difficult to be further degraded and may produce feedback inhibition on NHase. In view of the above reasons, the actual degradation rate of SXF in the field environment is usually only 1/5 to 1/20 of the laboratory measured value.

## 4 The degradation effect of NHase on SXF

### 4.1 The types and functions of key degradation enzymes in SXF

**FIGURE 4:**
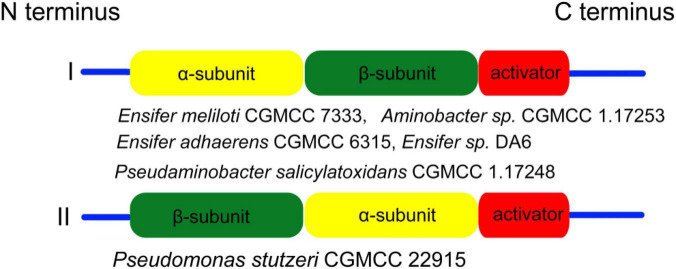
Composition of nitrile hydratase (NHase) gene reported for sulfoxaflor degradation.

SXF biodegradation is mechanistically driven by the hydration of its cyano group, which is the rate-limiting step and is exclusively catalyzed by nitrile hydratase (NHase). Other enzymes (P450, esterase) are involved in animal detoxification but not in environmental microbial degradation. NHase is the core enzyme for microbial degradation of SXF and has been studied most deeply. The cytochrome P450, esterase, glutathione reductase and other enzymes in animal bodies are involved in the detoxification process of SXF within the animals (Li et al., 2025; Ma et al., 2019; Wang et al., 2024; Xin et al., 2025). Cytochrome P450 catalyzes the *in vivo* biotransformation of SXF via phase I reactions (dimethyl sulfone stabilization, monooxygenation, demethylation, and hydroxylation) generating five phase I metabolites, which are further converted into four more hydrophilic phase II metabolites through sulfation, taurine conjugation, glutathione conjugation, and glucuronidation for detoxification and excretion (Xu et al., 2020). Carboxylesterases primarily contribute to SXF resistance in *Aphis gossypii* likely via sequestration, as indicated by elevated carboxylesterase activity and significant synergism of S, S, S-tributyl phosphorotrithioate in the resistant strain, despite SXG lacking a hydrolyzable carboxylic ester group (Ma et al., 2019). Glutathione S-transferases enhance detoxification against sulfoxaflor by increasing GST enzyme activity and upregulating gene expression, thereby mitigating oxidative stress induced by the insecticide (Balakrishnan et al., 2019). NHase, as a class of metal enzymes containing cobalt or iron ions, can specifically catalyze the hydrolysis of the carbonyl group (−CN) in the NEOs molecule to generate the amide group (−CONH_2_), which is the initial step and rate-limiting step of the degradation reaction (Sun et al., 2017; Zhou et al., 2014). The overall structure of these enzymes is diverse. In general, NHases involved in the degradation of NEOs and related insecticides can be classified into four typical types based on subunit organization and gene arrangement. Among them, only two types have been reported to degrade SXF, which are clearly illustrated in Figure 4.

### 4.2 Classification and structural characteristics of SXF-degrading NHases

NHase mediating SXF biodegradation are predominantly classified as cobalt-containing enzymes, characterized by α-subunit, β-subunit, and accessory protein components with diverse gene arrangements across bacterial strains. Most well-characterized SXF-degrading NHases follow the <α-subunit><β-subunit><accessory protein> gene order, such as those from *Aminobacter* sp. CGMCC 1.17253 (Yang et al., 2020), *E. meliloti* CGMCC 7333 (Sun et al., 2016), and *Ensifer* sp. DA6 (Yang et al., 2025). *P. stutzeri* CGMCC 22915 harbors a unique NHase with a reversed <β-subunit><α-subunit><accessory protein> gene orientation, representing the first reported such arrangement for SXF degradation (Jiang et al., 2022). *P. salicylatoxidans* CGMCC 1.17248 possesses two functional NHases (AnhA and AnhB) encoded on plasmid and chromosome, respectively, both capable of SXF hydration with distinct catalytic efficiencies (Guo et al., 2021). *S. canus* CGMCC 13662 features a novel three-subunit NHase (AnhD, AnhE, AnhA) without an accessory protein, with AnhD and AnhE homologous to the N- and C-terminal fragments of typical β-subunits (Guo et al., 2019). These enzymes share conserved cobalt-binding motifs, including key residues such as αCys-105, αCys-108, and αCys-110 in *Aminobacter* sp. NHase (Yang et al., 2020), αCys-118, αCys-121 in *P. salicylatoxidans* AnhA, and αCys-107, αCys-110, αCys-112 in AnhB (Guo et al., 2021). Residues like βArg52 (AnhA/AnhB) and βHis21 (AnhE) contribute to substrate binding and catalytic efficiency (Guo et al., 2019; Jiang et al., 2022). The eukaryote-derived *Monosiga brevicollis* NHase, with a fused α/β-subunit structure and histidine-rich region, parallels the structural diversity of prokaryotic SXF-degrading NHases. Phylogenetic analysis shows SXF-degrading NHases cluster with NHases from *Sinorhizobium*, *Rhizobium*, and *Agrobacterium*, while *S. canus* CGMCC 13662 NHase forms an independent branch with *Streptomyces rimosus* NHase (Sun et al., 2021b). Since no experimentally resolved crystal structure is available for SXF-degrading NHases, their overall structures are highly similar to known Co-type NHases with a typical α/β heterodimer fold and conserved Co-binding active site. High-confidence structural predictions can be further constructed using AlphaFold2 in future studies to reveal detailed catalytic mechanisms.

### 4.3 Key influences on catalytic activity of NHase

The catalytic activity of SXF-degrading NHases is dynamically regulated by environmental factors, structural features, and chemical modulators, with notable variations among strains. Among characterized NHases, *P. salicylatoxidans* AnhA has the highest catalytic efficiency with Vmax = 4.4 U mg^1^ min^1^ and Km = 0.95 mmol/L. *Aminobacter* sp. NHase shows lower efficiency (Vmax = 1.97 U/mg, Km = 3.54 mmol/L). No NHase can achieve complete mineralization of SXF to CO_2_ and NH_3_ (Zhao et al., 2023).

#### 4.3.1 Environmental factors

Optimal pH ranges from 6.0 to 8.0. *P. stutzeri* PsNHase exhibits maximum activity at pH 6.0 (Jiang et al., 2022), *Aminobacter* sp. NHase at pH 7.0 (Yang et al., 2020), and *P. salicylatoxidans* AnhB maintains > 95% activity across pH 5.0–9.0 (Guo et al., 2021). *S. canus* CGMCC 13662 NHase retains > 95% activity at pH 5.0–9.0, showing broader pH stability (Guo et al., 2019). Optimal temperatures span 30–50 °C. PsNHase performs best at 30 °C (Jiang et al., 2022), AnhA from *P. salicylatoxidans* at 50 °C (Guo et al., 2021), and *S. canus* CGMCC 13662 NHase at 30 °C (Guo et al., 2019). Most NHases are unstable above 40–50 °C, with *Aminobacter* sp. NHase losing activity completely at 70 °C (Yang et al., 2020). Metal ions such as Co^2+^, Mg^2+^, and Mn^2+^ enhance activity by facilitating cobalt incorporation (Guo et al., 2021; Yang et al., 2020), whereas Cu^2+^ and Ag^+^ strongly inhibit most NHases by disrupting metal coordination (Jiang et al., 2022; Zhao Y.-X. et al., 2025). AnhB shows high tolerance to Zn^2 +^, Mn^2 +^, and Ni^2+^ (Guo et al., 2021). Organic solvents (e.g., ethanol, ethyl acetate) generally suppress activity, with *P. salicylatoxidans* AnhB retaining > 92% activity and *S. canus* CGMCC 13662 NHase showing slight inhibition by dichloromethane (Guo et al., 2019, 2021).

#### 4.3.2 Structural and chemical modulators

Substrate inhibition occurs at high SXF concentrations (> 1,000 mg/L) for most enzymes (Jiang N. et al., 2023). Key Structural Residues: β-His62 in PsNHase, β-Arg52 in AnhA, and AnhD-Glu56/AnhE-His21 in *S. canus* NHase contribute to substrate binding and catalytic efficiency (Guo et al., 2019, 2021; Jiang et al., 2022). *Aminobacter* sp. NHase has a Km of 3.54 mmol/L and Vmax of 1.97 mU/mg (Yang et al., 2020). *P. salicylatoxidans* AnhA exhibits higher affinity (Km = 1.02 mmol/L) and catalytic efficiency (Vmax = 14.42 μmol mg^1^ min^1^) (Guo et al., 2021). *S. canus* CGMCC 13662 NHase has a Km of 5.85 mmol/L and Vmax of 15.99 U/mg (Guo et al., 2019). The general nitrogen regulation protein NtrC bidirectionally regulates nitrile hydratase expression in *E. adhaerens* CGMCC 6315: it induces nitrile hydratase expression under ammonium-limited conditions while repressing it when ammonium is sufficient (Jiang N. et al., 2023). Nutrient deprivation upregulates the expression of the nitrile hydratase gene pnhA in *E. adhaerens* CGMCC 6315, significantly enhancing its nitrile hydratase activity and acetamiprid degradation efficiency, while having no obvious effect on the expression of the other nitrile hydratase gene cnhA (Sun et al., 2018).

### 4.4 Biotechnological optimization strategies

Heterologous expression and immobilization have emerged as pivotal strategies to enhance NHase applicability in SXF bioremediation, with advancements in genetic engineering and enzyme stabilization.

#### 4.4.1 Heterologous expression of NHase

NHase genes from various strains are successfully expressed in *E. coli* Rosetta (DE3). *E. coli* expressing PsNHase degrades 90.4% of SXF within 5 min (Jiang et al., 2022). *E. coli* harboring *Pseudaminobacter salicylatoxidans* AnhA shows high substrate affinity (Km = 1.02 mmol/L) (Guo et al., 2021). *E. coli* expressing *S. canus* CGMCC 13662 NHase degrades 83.1% of SXF in 10 min (Guo et al., 2019). Co-expression of cobalt transporter genes (e.g., PsCbiM) improves NHase activity by 2–4 folds via enhanced cobalt uptake (Jiang H. et al., 2023). Disrupting overlapping sequences with a strong ribosome-binding site (RBS) enhances Variovorax boronicumulans NHase activity (Sun et al., 2016).

#### 4.4.2 Immobilization techniques

Calcium alginate, sodium alginate-polyvinyl alcohol (SA-PVA), or Ni-NTA resin enhance stability and reusability. Immobilized *P. stutzeri* cells retain 72.9% activity after 60 days (Jiang et al., 2022). Immobilized *E. coli* cells expressing PsNHase-PsCbiM maintain 42.2% activity after 6 days of storage (Jiang H. et al., 2023). Cell-immobilized NHase from E. adhaerens efficiently degrades SXF in soil and surface water, with a degradation rate of 89.7% in co-contaminated environments (Yang et al., 2025). *Aminobacter* sp. CGMCC 1.17253 degrades 59.1% of SXF in soil within 9 days (Yang et al., 2020), and *P. salicylatoxidans* CGMCC 1.17248 eliminates SXF residues in soil within 4 days (Guo et al., 2021).

## 5 Conclusions and perspectives

### 5.1 Main conclusions

As a pivotal substitute for traditional NEOs in modern agriculture, SXF effectively controls piercing-sucking pests but faces sustainability challenges due to its environmental persistence, migration potential, and the toxic risks of metabolites like X11719474. Significant progress has been made in understanding SXF’s environmental fate including its migration, transformation, and residue characteristics in water bodies, soils, and agricultural products, identifying degrading microorganisms (e.g., *Ensifer*, *Pseudomonas*, *Aminobacter*, and cyanobacteria such as *Synechocystis salina*) and deciphering NHase-mediated catalytic mechanisms. Key findings confirm that NHase serves as the core enzyme in SXF biodegradation, with Co-type NHases being predominant among degrading strains, exhibiting diverse gene arrangements, structural characteristics, and catalytic properties that are dynamically regulated by environmental factors (pH, temperature, metal ions), structural residues, and chemical modulators. Biotechnological strategies such as heterologous expression and immobilization have been validated to enhance NHase’s solubility, activity, and reusability in SXF remediation. However, critical gaps remain such as incomplete cross-media migration data of SXF and its secondary metabolites in complex agricultural ecosystems, limited diversity of degrading microbes (especially from extreme environments), unclear regulatory networks of microbial degradation pathways, and technical bottlenecks in NHase’s industrial application (e.g., narrow adaptability, low recombinant solubility, incomplete mineralization). This review systematically integrates SXF’s environmental behaviors, degrading microbial resources, key enzymatic mechanisms, and biotechnological optimizations, providing comprehensive theoretical support for the scientific application and environmental risk control of SXF, and offering references for the biodegradation research of novel neonicotinoid-like insecticides.

### 5.2 Perspectives

Critical gaps remain such as incomplete cross-media migration data of SXF and its secondary metabolites in complex agricultural ecosystems, limited diversity of degrading microbes (especially from extreme environments), unclear regulatory networks of microbial degradation pathways, and technical bottlenecks in NHase’s industrial application (e.g., narrow adaptability, low recombinant solubility, incomplete mineralization). Future research should prioritize systematic cross-media fate and ecological risk assessments using multi-isotope tracing and high-resolution mass spectrometry, expand degrading microbial resources via metagenomics, integrate multi-omics to clarify degradation regulatory networks, optimize NHase performance through enzyme engineering and novel immobilization technologies, and strengthen field validation of bioremediation products. By addressing these gaps through interdisciplinary collaboration, we can advance efficient, low-cost bioremediation solutions, ensure food and ecological security, and provide a blueprint for the sustainable use of SXF and biodegradation research of novel neonicotinoid-like insecticides, fostering the green development of modern agriculture.

## Footnotes

1
https://www.mda.state.mn.us/sites/default/files/2019-10/sulfoxaflorreview.pdf
